# Understanding refugee and immigrant health literacy and beliefs toward antimicrobial resistance

**DOI:** 10.1017/ash.2023.443

**Published:** 2023-10-18

**Authors:** Joseph Ladines-Lim, Elizabeth Scruggs-Wodkowski, Tessa Adžemović, Rachel Croxton, Ron Romero, Michael Lukela, Krishna Rao, Preeti Mehrotra, Payal K. Patel

**Affiliations:** 1 Departments of Internal Medicine and Pediatrics, University of Michigan, Michigan Medicine, Ann Arbor, MI, USA; 2 Division of Infectious Diseases, University of Michigan, Michigan Medicine, University Hospital South F4012A, Ann Arbor, MI, USA; 3 University of Michigan Medical School, Ann Arbor, MI, USA; 4 Packard Health, Ann Arbor, MI, USA; 5 Silverman Institute for Health Care Quality and Safety and Division of Infectious Diseases, Beth Israel Deaconess Medical Center, Boston, MA, USA; 6 Intermountain Medical Center – Infectious Disease, Murray, UT, USA

## Abstract

Refugee and migrant populations have increased vulnerability to antimicrobial resistance, yet stewardship guidance is lacking. We addressed this gap through a cross-sectional survey, finding that these populations and immigrants from low and middle-income countries had lower health literacy on the issue compared to native-born Americans and those from high-income countries.

## Introduction

Antimicrobial resistance (AMR) is a global health threat with higher prevalence in migrant and refugee populations,^1,2^ yet public health agencies have provided little guidance on stewardship interventions to counter this threat.^3^ Past research has focused on encampment areas or other countries abroad with little attention in the United States (US). Furthermore, poor health literacy regarding antibiotic use has been shown to be associated with inappropriate antibiotic use,^4,5^ which contributes to AMR and is hence a key research need we have highlighted previously in refugee populations.^3^

Therefore, we aimed to assess health literacy in appropriate antibiotic use and AMR in patients with history of refugeeism, asylum-seeking, or immigration from low and middle-income countries (LMICs) in the US. We hypothesized that this target population had gaps in health literacy compared with native-born Americans and those from high-income countries (HICs). Regarding health literacy, we focused on appropriate antibiotic use in the context of pediatric respiratory infections, a setting in which inappropriate antibiotic use is common.^5^

## Methods

From November 1, 2022 to March 10, 2023, we implemented a cross-sectional 17-question anonymous survey derived from a previously validated questionnaire that focused on pediatric respiratory infections, a common problem in stewardship.^5,6^ We used convenience sampling, distributing this on paper to anyone 18 years or older at three primary care clinics in Southeastern Michigan with generally higher proportions of refugee/asylee/immigrant populations. Other participants could access the same survey electronically through Qualtrics and umhealthresearch.org (https://UMHealthResearch.org/#studies/HUM00216788), a Michigan Medicine website that allows anonymous users to participate. To incentivize participation, we offered in-person participants an optional gift card of modest value ($5); due to technical constraints, we did not offer this online.

We collected demographic information, including history of past refugeeism/asylum-seeking/immigration, country of origin, age group, duration of residence in the US, gender, educational level, English ability, and income (categorized as below 100% of federal poverty level for household of four, between 100% and 185%, and above 185%).^7^ Based on responses, we categorized participants into three groups: former refugees/asylees, non-refugee/asylee immigrants from LMICs (“Immigrant-LMIC”), or those from the US or HICs (“US/HIC”) as classified by the World Bank.

We assessed health literacy using eight 5-point Likert scale questions on pediatric respiratory infections given known inappropriate antibiotic use in this setting.^5^ Because clinics requested we minimize survey burden for patients due to time constraints, we included only questions that could reasonably fit on one page, omitting others.^6^ We provided verified translations of the questionnaire in Albanian, Amharic, Arabic, Burmese, Cebuano, Dari, Farsi, French, Hausa, Hindi, Kinyarwanda, Pashto, Portuguese, Punjabi, Russian, Somali, Spanish, Swahili, Tagalog, Tigrinya, Ukrainian, Urdu, Vietnamese, and Yoruba. The Institutional Review Boards of the University of Michigan Medical School and Trinity Health deemed the study exempt.

We used χ^2^ and Wilcoxon–Mann–Whitney tests where appropriate with *p*-values less than 0.05 significant. To account for likely covariance between Likert scales and avoid the multiple comparisons problem, we derived an overall health literacy score from binarized Likert scale responses. We assigned 1 point for preferred responses and 0 points otherwise, summing these to generate the overall score (maximum of 8 points). Treating site of data collection as a randomized variable in a mixed model, we used multivariable linear regression to identify variables independently associated with the overall health literacy score, with p-values less than 0.1 significant. We treated variables with more than two categories as ordinal rather than continuous to avoid assumptions of a continuous linear association with the health literacy score. We excluded any surveys lacking any response from regression. We used R version 4.2.3 (R Foundation for Statistical Computing, Vienna, Austria) for analysis.

## Results

Our study had 280 participants from 40 different countries, with 18 in the “Former refugees/asylees” group, 92 in the “Immigrant-LMIC” group, and 170 in the “US/HIC” group. Given the first group’s limited size and statistical power, as well as the general similarity in demographics between the first two groups (Table 1), we aggregated these into a “Refugees/asylees/immigrant-LMIC” group for comparison with the “US/HIC” group. Participants answered most questions with the lowest completion rate being the “Former refugees/asylees” group and household income question at 72%.

**Table 1.** Demographic characteristics of former refugees/asylees, former immigrants from low and middle-income countries (“Immigrant-LMIC”), and those from the United States and high-income countries (“US/HIC”). Data for aggregation of former refugees/asylees and the immigrant-LMIC group, designated as the “Refugees/asylees/immigrant-LMIC” group, are also shown. All data are expressed as percentages. Total sample sizes for each group are shown in parentheses in the top row. Total number of responses for each demographic variable/group is also shown throughout. *P*-values reflect χ^2^ or Wilcoxon–Mann–Whitney tests where appropriate

	Former refugees/asylees	Immigrant-LMIC	Former refugees/asylees versus Immigrant-LMIC	Refugees/asylees/immigrant-LMIC	US/HIC	Refugees/asylees/immigrant-LMIC versus US/HIC
	(*N* = 18)	(*N* = 92)	(*p*-value)	(*N* = 110)	(*N* = 170)	(*p*-value)
Female gender	50.0	64.0	0.452	61.9	69.7	0.198
	(*N* = 16)	(*N* = 89)		(*N* = 105)	(*N* = 162)	
Age (years)	*N* = 18	*N* = 80	0.288	*N* = 98	*N* = 165	0.843
18–24	0	5.0		4.1	4.8	
25–34	38.9	22.5		25.5	18.2	
35–44	27.8	16.3		18.4	19.4	
45–54	5.6	11.3		10.2	21.2	
55–64	5.6	20.0		17.3	18.2	
65–74	16.7	17.5		17.3	9.7	
75–84	5.6	6.3		6.1	8.5	
> 80	0	1.3		1.0	0	
Duration of residence in the United States (years)	*N* = 16	*N* = 89	0.010	*N* = 100	*N* = 167	<0.001
0–10	93.8	58.6		64.1	1.2	
10–20	0	13.8		11.7	1.2	
20–30	6.3	20.7		18.4	14.5	
30–40	0	2.3		1.9	18.1	
40–50	0	3.4		2.9	21.1	
50–60	0	1.1		1.0	19.3	
60–70	0	0		0	12.0	
70–80	0	0		0	9.0	
> 80	0	0		0	3.6	
Income	*N* = 14	*N* = 69	0.888	*N* = 83	*N* = 137	<0.001
< 100% poverty level	85.7	69.6		72.3	16.1	
100–185% poverty level	7.1	18.8		16.9	18.2	
> 185% poverty level	7.1	11.6		10.8	65.7	
Educational level	*N* = 18	*N* = 91	0.521	*N* = 109	*N* = 167	<0.001
No formal schooling	0	7.7		6.4	0.6	
Primary schooling	22.2	16.5		17.4	7.8	
Secondary schooling	44.4	27.5		30.3	7.8	
College/University	22.2	28.6		27.5	40.1	
Graduate degree	11.1	19.8		18.3	43.7	
English ability	*N* = 13	*N* = 88	0.048	*N* = 101	*N* = 167	<0.001
No knowledge	46.2	27.3		29.7	0.6	
Elementary knowledge	30.8	22.7		23.8	1.8	
Limited working knowledge	15.4	17.0		16.8	2.4	
Professional working knowledge	7.7	17.0		15.8	9.0	
Fluent/native speaker	0.0	15.9		13.9	86.2	

**Figure 1.**
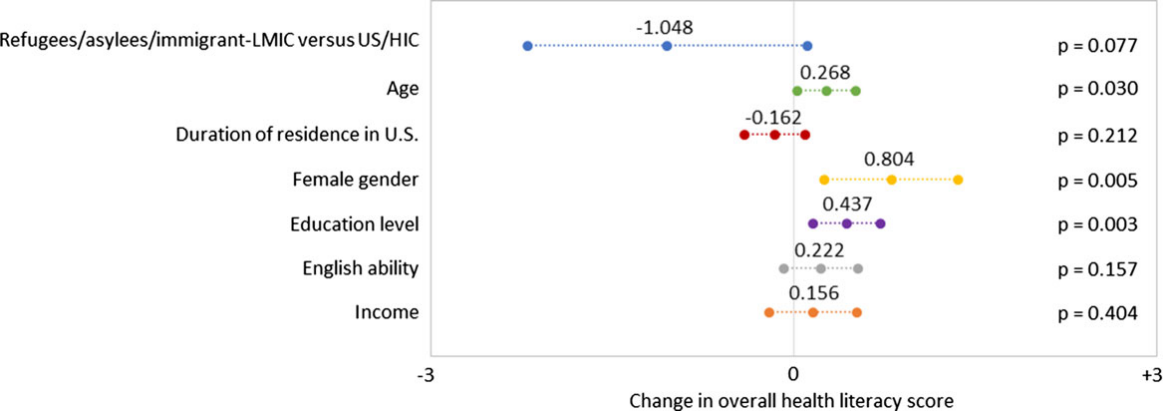
Estimated effect size of demographic variables on overall health literacy score for the refugee/asylee/immigrant-LMIC and US/HIC groups derived from multivariable linear regression. The “Refugee/asylee/immigrant-LMIC” group included participants who identified as former refugees, asylees, or immigrants from low or middle-income countries. The “US/HIC” group included participants who identified as from the United States or high-income countries. World Bank classifications were used for country income status. A positive change in the overall health literacy score indicated more clinically preferred responses while a negative change indicated fewer. End points of lines represent lower and upper limits of 95% confidence intervals.

Age distribution did not differ between the two main groups, while duration of residence in the US did with most of the “Refugees/asylees/immigrant-LMIC” group having resided between 0 and 10 years (Table 1), nearly half between 0 and 2 years. We found significant differences in other demographics except for gender proportion, with the “Refugees/asylees/immigrant-LMIC” group reporting higher prevalence of poverty, lower education, and lower English ability (Table 1). Excluding 35% of the surveys due to at least one missing response, multivariable linear regression revealed that female gender, educational level, age, and the “US/HIC” group compared to the “Refugees/asylees/immigrant-LMIC” group were independently associated with increased overall health literacy (Figure 1).

## Discussion

Our study showed former refugees/asylees/immigrants from LMICs had gaps in knowledge of appropriate antibiotic use and AMR compared to native-born Americans or those from HICs. While other related research has been conducted in various encampment areas abroad,^3^ our study is unique in its assessment of this population resettled in the US. Most strikingly, history of refugeeism/asylum-seeking/immigration from an LMIC most strongly correlated with lower health literacy, independent of other demographic variables. This may be due to other factors our survey did not capture that are likely significant components of the immigrant/refugee experience. Given the association between health literacy in this domain and inappropriate antibiotic use established in past work,^4,5^ our findings suggest this population is at increased risk of inappropriate antibiotic use promoting AMR.

Interestingly, other variables independently correlated with greater health literacy, including female gender, age, and educational level. While education intuitively correlates with health literacy, we did not expect female gender and age necessarily to do so, though the latter’s effect size was small. Female gender had a more pronounced effect, a unique though plausible finding given past work demonstrating gendered differences regarding views on AMR due to traditional cultural expectations for childcare.^8^ Regardless, we feel this requires further investigation and agree with the World Health Organization’s call for a focus on gender in combatting AMR.^9^

We acknowledge our study’s limitations. While our sample size enabled statistically meaningful comparisons, it is still a fraction of our target populations, meaning a larger sample size could reveal different trends. Sample sizes of individual countries (except the US) and geographic regions were also limited, precluding any comparative analysis. We also did not address all possible social and demographic factors that could plausibly impact health literacy, such as number of household children, due to the desire to minimize survey burden on participants. There was also some degree of bias due to incomplete responses, recall bias, and selection bias given our decision to make the survey available to the general public online.

Nonetheless, we hope our findings will be useful in producing an educational intervention with translations to native languages so as to overcome potential cultural barriers that have hindered past stewardship efforts,^10^ such as mistrust of the US providers.^4^ This is currently the next phase of our work in the target population, which, if shown to improve understanding of appropriate antibiotic use and related outcomes, could serve as a template for future interventions regarding antimicrobial stewardship in refugees and immigrants, particularly those recently resettled in the US.

## Supporting information

Ladines-Lim et al. supplementary material Ladines-Lim et al. supplementary material

## Supplementary material

For supplementary material accompanying this paper visit http://doi.org/10.1017/ash.2023.443. click here to view supplementary material
